# Public vs. private job dilemma: Influencing factors in career selection for university graduates

**DOI:** 10.1371/journal.pone.0258331

**Published:** 2021-10-11

**Authors:** Sadia Sharmin Suhi, Ferdousi Jahan Oyshi, Md. Abdulla Al Mamun, Nusrat Jahan, Tunvir Ahamed Shohel, Molla Azizur Rahman, Md. Nazrul Islam, Md. Tanvir Hossain

**Affiliations:** 1 Sociology Discipline, Social Science School, Khulna University, Khulna, Bangladesh; 2 Soto Angtihara Sundarban Adarso Government Primary School, Koyra, Khulna, Bangladesh; 3 English Discipline, Arts and Humanities School, Khulna University, Khulna, Bangladesh; 4 Forestry and Wood Technology Discipline, Life Science School, Khulna University, Khulna, Bangladesh; Neijiang Normal University, CHINA

## Abstract

The unprecedented growth of educated workforce following the economic development and diversity in workplace has widened the career choices of young people in Bangladesh. However, it intensifies a dilemma among the job seekers about determining their career goals, because career goals are often influenced by certain socio-demographic and cultural aspects. Hence, this cross-sectional study was designed to investigate the career choices of university students in Bangladesh and to identify its determinants. Administering a self-administered questionnaire (SAQ), data were collected from 422 students at a public university using the multistage stratified sampling. Data were analyzed by bivariate (chi-square) and multivariate (exploratory factor analysis and binary logistic regression) analyses. Findings suggest that the career choices of students vary regarding their gender, religion, and academic track. For example, female (AOR: 0.281; 95% CI: 0.144 to 0.547) and Muslim (AOR: 3.648; 95% CI: 1.765 to 7.542) students preferred public jobs, whereas students of commerce (AOR: 0.344; 95% CI: 0.144 to 0.820) went for private ones. Among socioeconomic issues, only the father’s occupation had a substantial effect on career decisions (AOR: 0.347; 95% CI: 0.144 to 0.820). The career choice was also determined by the job prospects (AOR: 1.251; 95% CI: 1.161 to 1.347), preference of family (AOR: 1.238; 95% CI: 1.099 to 1.394), as well as job diversity (AOR: 0.879; 95% CI: 0.795 to 0.972). Based on the findings of this study, it is recommended that the government should address the trends and patterns of career choices of students through empirical research when formulating future educational and career-related policies in Bangladesh.

## Introduction

After the Liberation War in 1971, Bangladesh made education one of its top priorities to develop a holistic approach of progress through optimum utilization of skilled and dedicated workforce [1]. The succeeding governments–both civilian and military–invested the financial and human resources, though insufficient [2], to ensure inclusive and quality education for all in consonance with the first education commission report [3]. Subsequently, Bangladesh has been experiencing impressive growth in the enrolment as well as completion rate with increased gender parity at all levels of education in the last three decades [3–5]. Despite the economic growth, the employment opportunities have shrunk disproportionately, considering the growth of the population and the educated workforce over the past decade [6–9]. For females alone, unemployment has increased from 1 million in 2010 to 1.2 million in 2018 [10], while the overall unemployed people stood around 2.7 million, of which 15% were university graduates [9]. The supply and demand gaps, thereby, inevitably produced an extreme competition for scarce job opportunities. A recent report suggests that more than 6 million people have submitted their application against a mere 23,616 government jobs in 2018–2019 [11].

In Bangladesh, the growing demand for government jobs is relatively a recent phenomenon. A youth survey in August 2018 suggested that government job has become a prominent life goal for most of the young, educated people in Bangladesh [12]. The increased financial incentives together with job security, structured retirement plan as well as the failure of private sectors to create new employment opportunities are contributing to the growing demand for government jobs in Bangladesh [10, 11, 13]. In contrast to Bangladesh, it is evident that more people in developed, and newly industrialized countries (NICs) are more interested in private sectors [14, 15]. Because, private sectors provide better financial schemes, in terms of monthly and hourly wages [14, 16], although workers in private sectors are bound to for work longer hours, and they are comparatively dissatisfied with their working environment [14, 15].

An individual’s career choices are influenced by a range of factors, such as personal characteristics, socioeconomic background, social and cultural expectations, and preferences. Studies showed that educational self-efficacy, enthusiasm for community betterment and financial returns along with personal characteristics, i.e., gender, academic grades, and socioeconomic status (SES) and family expectations significantly influenced the career choice [17–19]. Besides, geographical location and cultural variation also determine the career choice of individuals [20–22]. For example, the Asian students were more likely to select a career due to the influence of parents and peers, as well as financial returns [22, 23]. In contrast, the African American women had more career aspiration, influenced by knowledge and commitment, compared to their Caucasian compatriots [21].

In Bangladesh, there is none but a few empirical studies on career choice [12, 24]. That too, however, did not point out the determinants of career choices of the university students. Hence, this cross-sectional study was designed to identify career choices of the university students and the factors influencing career choice decisions. Considering Homans’ [25] view of fair exchange theory as well as Coleman’s [26] interpretation of social capital, this study, therefore, attempted to find out the answers to two research questions, what are the jobs preferred by the students at the university in Bangladesh? And what are the factors–social, economic, or other related issues–influencing their career choices? This study will help the policymakers–of both public and private sectors–to comprehend the trends and patterns of career choices made by the young Bangladeshi people and to implement policies to prepare skilled workforce to meet the pre-requisites of the twenty-first century.

## Theoretical background

In explaining human actions, especially economic activities to satisfy ‘needs’ and pursue individual ‘goals’, Homans [25] introduced the economic concepts of ‘costs’ and ‘benefits,’ and argued that individual’s actions depend on the reward. Being rational, humans prefer ‘pleasure’–the remuneration–in return for their activities and intend to avert ‘pain’–the penance. In social settings, individuals always evaluate the costs and the return–the benefits–considering the standards set by society, while the standards are subject to persons, times, and spaces [27]. To maximize benefits or advantages, individuals by nature perform the rewarding actions repetitively. This study further incorporated Coleman’s [26] idea of social capital. For Coleman [26], the interpersonal social interactions and networks are subject to persistent social ‘benefits.’ The social capital, time and efforts spent by parents, together with financial (monetary) and human (educational attainment) capitals of parents play a significant role in the intellectual development of children that, in turn, shape the socioeconomic status (SES) of the latter.

From the theoretical understanding of Homans [25], it is articulated that individuals think critically about their perceived ‘costs’ and ‘benefits’, including social, economic, and cultural issues, when selecting a career. Kobia-Acquah, Owusu [18], for instance, found that optometry students in Ghana chose their career considering the benefits, including potential income and flexible working hour, while Rispel, Ditlopo [17] observed that health professionals in South Africa, when selecting a career, emphasized on the betterment of community through humanitarian activities as well as job security and good payment. Others, however, suggest that future career prospects and opportunities, including prestige, promotion as well as financial/monetary incentives, and working conditions, such as working hours and flexibility, are the prime determinants of career choices of individuals [19, 28–30].

Both Homans [25] and Coleman [26] informed that family among other issues plays an important role in making career choices. For example, both the demand and supply side of the family, e.g., informational, financial, and emotional support for family members, influence the career orientation of individuals [31–34]. The balance between work and family, i.e., women who are bound for their domestic roles, also found to be an inducing factor for individuals when deciding a career [32, 35, 36]. Besides, parental SES also determine the career aspiration of an individual. Individuals from low SES families generally have lower career aspirations [17, 20], whereas individuals from highly educated and affluent families are expected to ‘fit in the shoes’ of a ‘prestigious’ career of their parents [20, 37, 38]. The role of teachers as well as peer groups, to select a career, cannot be underestimated as well [17, 19].

In addition, the theories of Homans [25] and Coleman [26] also conceptualize the significance of personal characteristics in determining the perceived benefits of a career. Studies on career choice identified the role of personal characteristic, including age, i.e., young, and old select different jobs [17, 20], gender, e.g., men aspire for prestigious, highly paid and challenging jobs compared to women [19, 39, 40], religion (emphasizing on ‘honesty and integrity’ when selecting a job without being ‘immoral’) [21, 41] and educational track, including training and commitment for a specific job [39, 42], in deciding a career pathway.

**Fig 1:**
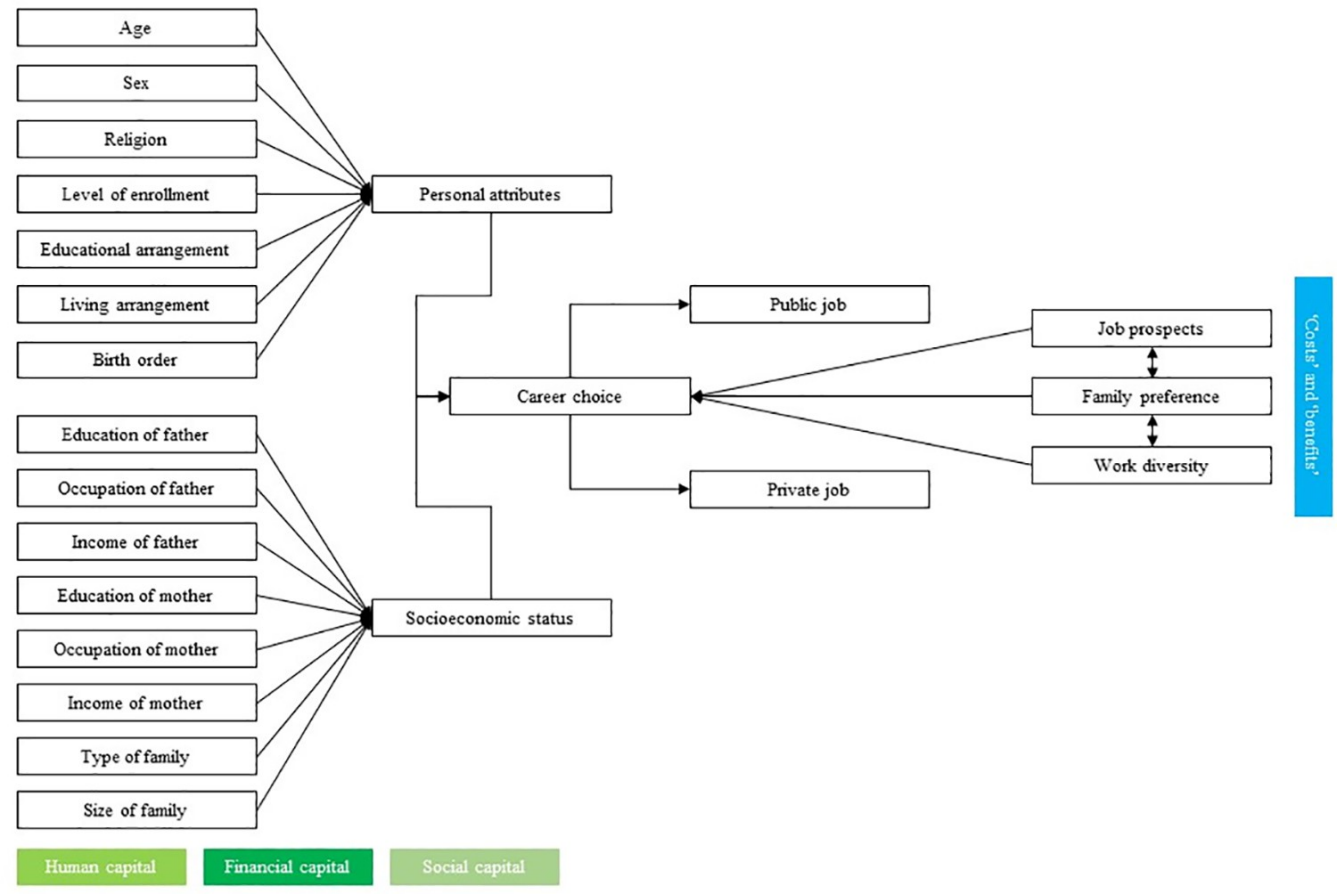
Conceptual framework based on the exchange theory of Homans [24] and the social capital theory of Coleman [25].

There are, indeed, numerous studies addressing the determinants of career choices across the world, and several factors–personal, social, economic, or academic–have been explored. However, a shortage of relevant literature in Bangladesh, especially at the tertiary level, is evident. This study, therefore, addressed the void in the existing literature, in conjunction with Homans’ fair exchange as well as Coleman’s social capital perspectives (see Fig 1 of the conceptual framework), to find out the factors influencing the career choices of university students in Bangladesh. This study is intended to attract the policymakers in higher academia of Bangladesh to re-think the underrepresented social issues in designing and enriching the educational environment as well as a professional specialization.

## Materials and methods

### Study site

**Fig 2:**
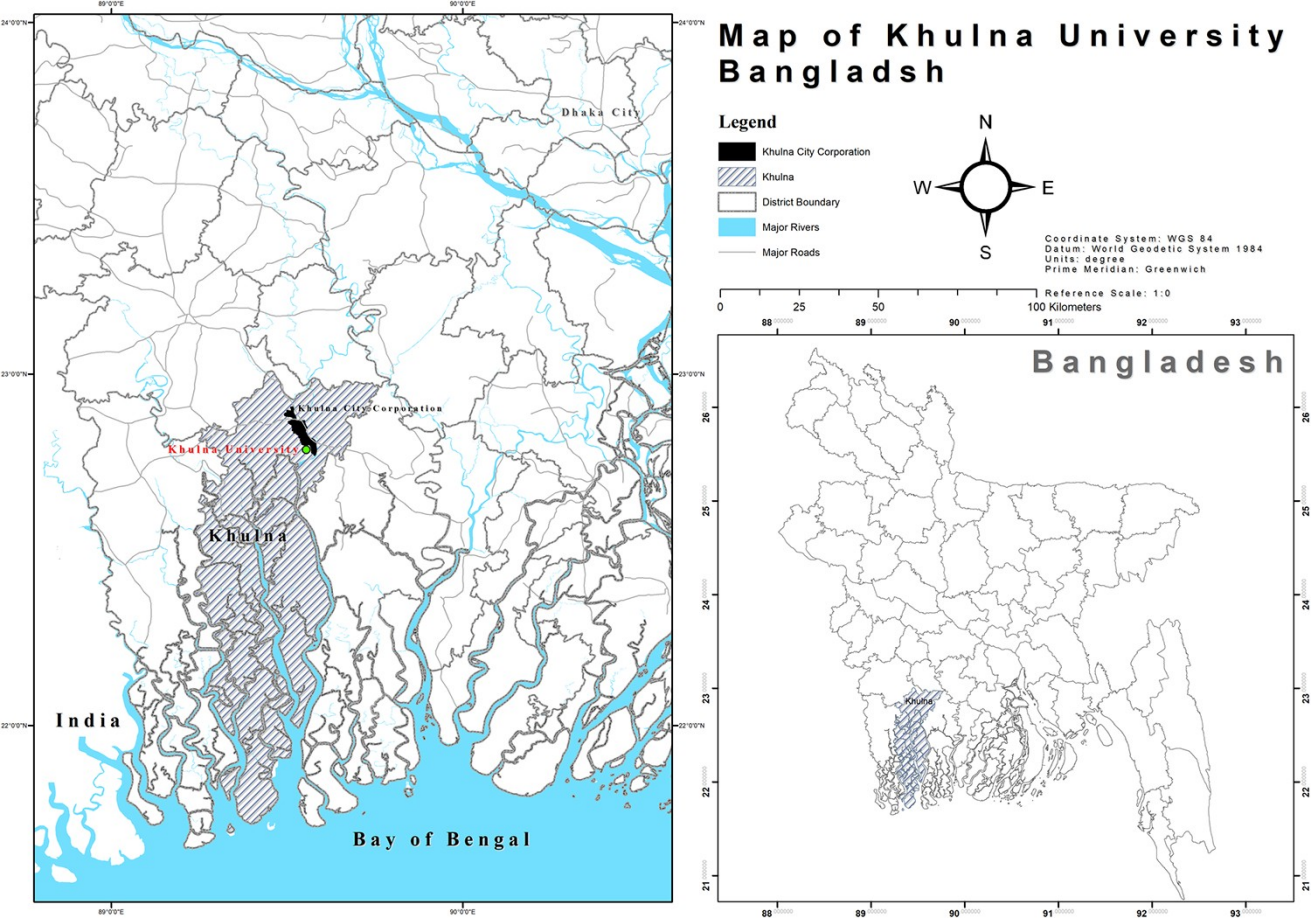
Study area.

The current study was carried out in Khulna University, a public university located in Khulna district of the southwestern region of Bangladesh (See Fig 2 of the study area). The university, starting the academic programs in late 1991 with 80 students in four Disciplines, has 28 Disciplines under six Schools and a single Institute. Out of 5,776 students, about 80% were enrolled in undergraduate programs, and of the total number, 59% of the students were male. The university represents an excellent example of diversity both in academic aspects and socioeconomic background of the students.

### Data

For this study, some specifications have been made to recruit participants: (i) the participants must be enrolled in regular programs, and (ii) they must not be a grade/term repeater (having backlog or retake course) or not be suspended from academic activities for academic cheating. Based on the criteria mentioned above, multistage stratified sampling was used to select the participants. Initially, a list of students, with or without backlog or retake and history of suspension, was provided by the Registrar of Khulna University following a formal application. Afterwards, the students were divided into three broad categories based on the track of education, i.e., arts and humanities, commers and science. There were 15 Disciplines under ‘science’ track followed by 11 Disciplines under ‘arts and humanities’ and two Disciplines under ‘commerce.’ Finally, students of each track of education, considering the number of Disciplines in each category, were randomly approached during the lunch break, and a total of 637 self-administered questionnaires (SAQ) were disseminated among students, out of which 435 students–with both verbal and written consents–provided their feedback (the response rate was 68.3%). From the returned SAQs, 13 were discarded due to nonresponse to a good number of questions; hence, this study finally included 422 participants. It is important to note that the sample size was determined by the sampling formula given by Cochran [43]. According to the formula, for a population of 6,000, the representative sample size would be 375, at 95% confidence interval with a confidence error of 5%.

### Ethical statement

This study was approved by the Ethical Clearance Committee of Khulna University, Bangladesh (Reference Number–KUECC– 2021/03/15). All the participants responded to the survey by an initial verbal consent followed by filling up a written informed consent letter in the first section of the SAQ. The participants were free to decline from the survey at any moment without prior justification.

### Measures

#### Outcome variable

The participants were requested to mark their future career plans from a list of occupations developed after reviewing the Bangladesh Labor Force Survey [7–9]. The participants stated more than 30 different professions, including ‘Teaching’ at various levels, ‘Business/Entrepreneurship’ of several categories, ‘Civil Service’ of different types, ‘Corporate Jobs’ at national and internal organizations, ‘Research and Development’ and so on. After the compare and contrast of the collected technical data, 12 major professional groups were sorted based on similarities, and later, categorized into two-broad spectrum–‘public/government’ and ‘private/non-government’ jobs. The outcome variable, in this paper, was expressed in a binary form (1 for public/government job and 0 for private/non-government job) considering the youth survey report of 2018 by BRAC [12].

#### Explanatory variables

Different personal and socioeconomic characteristics, including age, sex, religion, education, educational track, birth order as well as parental education, occupation and income and family size were considered as explanatory variables based on previous studies on career choices. This study also examined some other factors, such as living arrangements and type of family, to understand its role in selecting appropriate career by the participants in the context of Bangladesh. Furthermore, three additional factors, including job prospects, family preference, and work diversity, were explored and later added in the multivariate analysis to understand their role in selecting professional careers by university students in Bangladesh.

### Procedure

A semi-structured SAQ in English was developed to collect data from university students. The SAQ was divided into five interrelated and sequential sections. The first section required personal details of the participants, which included their age, sex, religion, year of schooling, Discipline, Schools, living arrangement, and birth order. The second and third sections asked information regarding the socioeconomic background and detailed information of the household, respectively, including parental education, occupation, income, marital status, head of the household as well as type and size of the family. In the fourth section, the participants were requested to report their preferred future job from a list of sectors. The fifth and final section contained 30 ‘five-point’ Likert-scale questions regarding the perceived factors determining their preference of career. The academic and research advisory committee, made of undergraduate and postgraduate faculty staffs of concerned Discipline as well as experts from other Schools/Faculties, reviewed the SAQ and research procedures and approved the study. A pre-test on 20 students from various Disciplines was carried out to assess the validity and reliability of the embedded question items in the SAQ. The participants in the pre-test, however, were excluded from the final data collection phase. Following some minor modifications to the SAQ, in question order as well as wording based on the pre-test, the data collection procedure began, with the approval of the advisory committee, in December 2017 and ended in February 2018.

### Data analysis

Data were analyzed using Statistical Package for the Social Sciences (SPSS) version 20.0 in different phases and by utilizing various analytical techniques considering the nature of data. For example, bivariate analysis, Pearson’s chi-square (χ^2^) as well as Yate’s continuity correction (χ^2^_Yates_), was used to find out the significant statistical relationship between the outcome and explanatory variables at the first stage. Exploratory factor analysis (EFA) was executed, in the second stage, to find out the factor structure of 30 ‘five-point’ Likert-scale items in a meaningful and effective manner. Finally, multivariate logistic regression was conducted by using variables that were statistically significant in the binary analysis together with the latent factors explored through EFA.

## Results

### Sample characteristics

Table 1 presents the association between career choices and the personal attributes as well as socioeconomic status of the participants. Findings indicate that only sex (*p* < 0.01), religion (*p* < 0.05), and educational track (*p* < 0.01), out of seven variables considered as personal attributes, were significantly associated with the career choices of the participants. In contrast, it is apparent that parental education (*p* < 0.05 for father, and *p* < 0.01 for mother), occupation (*p* < 0.01 for father, and *p* < 0.01 for mother), income (*p* < 0.01 for father, and *p* < 0.01 for mother) as well as the type *p* < 0.05) and size of family (*p* < 0.0) were all significantly associated with the career choices made by the participants.

**Table 1:** Career choices based on personal and family factors.

Variable			Career Choice		Test statistics	Effect size	*p* value
			Private *N* (%)	Public *N* (%)			
**Personal attributes**							
	***Age (in Year)***						
		≤ 20	81 (67.5)	205 (67.9)	0.001^**a**^	-0.004 ^**⸷**^	0.940
		≥ 21	39 (32.5)	97 (32.1)			
	***Sex***						
		Female	31 (25.8)	158 (52.3)	23.300^**a**^	-0.240** ^**⸷**^	0.000**
		Male	89 (74.2)	144 (47.7)			
	***Religion***						
		*Sanatan*	37 (30.8)	60 (19.9)	5.230^**a**^	0.118* ^**⸷**^	0.022*
		Islam	83 (69.2)	242 (80.1)			
	***Level of enrollment***						
		First	69 (57.5)	157 (52.0)	2.796^**b**^	0.081 ^**⸶**^	0.577
		Second	12 (10.0)	45 (14.9)			
		Third	10 (8.3)	31 (10.3)			
		Fourth	14 (11.7)	38 (12.6)			
		Fifth (MSc/MSS/MA)	15 (12.5)	31 (10.3)			
	***Educational track***						
		Arts and social science	34 (28.3)	154 (51.0)	18.170^**b**^	0.208** ^**⸶**^	0.000**
		Commerce	23 (19.2)	35 (11.6)			
		Science (engineering and life)	63 (52.5))	113 (37.4)			
	***Living arrangement***						
		Hall	45 (37.5)	99 (32.8)	1.001^**b**^	0.049 ^**⸶**^	0.606
		Boarding house	51 (42.5)	143 (47.4)			
		Family	24 (20.0)	60 (19.9)			
	***Birth order***						
		Others	68 (56.7)	176 (58.3)	0.037^**a**^	-0.015 ^**⸷**^	0.847
		First-born	52 (43.3)	126 (41.7)			
**Socioeconomic status**							
	***Education of father***						
		Primary (≤ Class V)	31 (25.8)	76 (25.2)	8.068^**b**^	0.138* ^**⸶**^	0.045*
		Secondary (Class VI–Class X)	17 (14.2)	75 (24.8)			
		Higher secondary (Class XI–Class XII)	30 (25.0)	49 (16.2)			
		Tertiary (≥ Class XIII)	42 (35.0)	102 (33.8)			
	***Occupation of father***						
		Unskilled/semi-skilled	24 (20.0)	117 (38.7)	13.598^**b**^	0.180** ^**⸶**^	0.001**
		Business	39 (32.5)	73 (24.2)			
		Public/private service	57 (47.5)	112 (37.1)			
	***Income of father (in BDT)***						
		≤ 20,000	54 (45.0)	186 (61.6)	18.354^**b**^	0.209** ^**⸶**^	0.000**
		20,001–40,000	35 (29.2)	85 (28.1)			
		≥ 40,001	31 (25.8)	31 (10.3)			
	***Education of mother***						
		Primary (≤ Class V)	32 (26.7)	56 (18.5)	13.439^**b**^	0.178** ^**⸶**^	0.004**
		Secondary (Class VI–Class X)	34 (28.3)	144 (47.7)			
		Higher secondary (Class XI–Class XII)	30 (25.0)	60 (19.9)			
		Tertiary (≥ Class XIII)	24 (20.0)	42 (13.9)			
	***Occupation of mother***						
		Housewife	92 (76.7)	274 (90.7)	13.558^**a**^	-0.187** ^**⸷**^	0.000**
		Working mother	28 (23.3)	28 (9.3)			
	***Income of mother (in BDT)***						
		No income	88 (73.3)	267 (88.4)	22.560^**b**^	0.231** ^**⸶**^	0.000**
		≤ 20,000	14 (11.7)	26 (8.6)			
		≥ 20,001	18 (15.0)	9 (3.0)			
	***Type of family***						
		Extended	27 (22.5)	101 (33.4)	4.363^**a**^	-0.107* ^**⸷**^	0.037*
		Nuclear	93 (77.5)	201 (66.6)			
	***Size of family (in Person)***						
		≤ 4	54 (45.0)	103 (34.1)	3.909^**a**^	0.102* ^**⸷**^	0.048*
		≥ 5	66 (55.0)	199 (65.9)			

^*.^ *p <* 0.05

^**.^ *p* < 0.01.

^**a.**^ Yate’s continuity correction

^**b.**^ Pearson’s chi-square.

^**⸷.**^ Phi (*φ*); ^**⸶.**^ Cramer’s V (*φ*_*c*_).

### Exploring career choice-related factors

The 30 five-point Likert-scale items were considered for EFA. The Kaiser-Meyer-Olkin measure statistic (0.698) and Bartlett’s test of sphericity (*p* < 0.001) verify the appropriateness of the samples for factor analysis. The EFA was conducted by executing the maximum likelihood extraction method using promax rotation. Three methods determined the decision of retaining factors–Kaiser’s criterion (eigenvalues 1) [44], a visual scree plot test [45] as well as parallel analysis [46]. The latter was used by comparing eigenvalues obtained from principal component analysis (PCA) with eigenvalues produced from a random data set of the same sample size, and the factors exceeding the values of randomized data were retained for analysis. The parallel analysis was executed by using software developed by Watkins [47]. Kaiser’s criterion suggested ten factors. It was, however, rejected for an inadequate number of items (< 3) with a poor pattern coefficient (≥ 0.30). In contrast, a visual inspection of the scree plot, as well as parallel analysis, suggested a three-factor solution, which met the criterion mentioned above, therefore, retained for analysis.

Based on the content of the items, the three-factor solution (with a Cronbach’s α = 0.756) suggested the following dimensions: job prospects, family preference, and work diversity (see Table 2). The first-factor ‘job prospects’–explaining 23.73% variance with a Cronbach’s α = 0.734 –comprised of 5 items that reflected the work-related facilities or opportunities generally considered by individuals to select an appropriate job. The second factor–consisted of 3 items and explaining 14.66% variance with an internal consistency of 0.605 –‘family preference’ appeared to reflect the family issues often prioritized by persons while selecting a career that offers sufficient spare time and space for family members. The third and final factor, ‘work diversity’, explained around 12.81% variance with a Cronbach’s α = 0.501, exclusively highlighted the thought-provoking properties of a work that often motivates workers to exploit their work-related mastery and skills.

**Table 2:** Exploratory factor analysis (N = 422).

Items		Pattern matrix			Structure matrix		
		Job prospects	Family preference	Work Diversity	Job prospects	Family preference	Work Diversity
2	Job security	0.858			0.801		
1	Salary	0.595			0.600		
4	Job incentive	0.594			0.597	0.476	
3	Job satisfaction	0.490			0.529		
15	Future safety & security	0.453			0.491		
11	Time for Family		0.712			0.678	
14	Family responsibility		0.613			0.597	
22	Working hour		0.549			0.543	
9	Travel opportunities			0.472	.0418		0.491
16	Social influence			0.458			0.463
29	Challenging & interesting			0.451			0.431
19	High social demand			0.410			0.427
**Variance explained**		**23.73**	**14.66**	**12.81**			
**Cronbach’s α**		**0.734**	**0.605**	**0.501**			

### Factors influencing career choices

A total of 15 variables was used in bivariate analysis, out of which eleven variables, including sex, religion, educational track, parental education, occupation, and income as well as type and size of the family found to have statistically significant relation with career choices made by the participants. The multivariate logistic regression was executed considering eleven significant variables from the chi-square analysis. Moreover, three additional factors, i.e., job prospects, family aspects, and work diversity, extracted from EFA, were also included in the regression analysis (see Table 3).

**Table 3:** Unadjusted and adjusted ORs for factors associated with career choice.

Factors			Unadjusted logistic regression model			Adjusted logistic regression model		
			COR	95% CI for COR	*p* value	AOR	95% CI for AOR	*p* value
**Personal attributes**								
	***Sex***							
		Female (ref)	1.000			1.000		
		Male	0.317	(0.199, 0.506)	0.000***	0.281	(0.144, 0.547)	0.000***
	***Religion***							
		Others (ref)	1.000			1.000		
		Islam	1.798	(1.113, 2.904)	0.017**	3.648	(1.765, 7.542)	0.004***
	***Educational track***							
		Arts and Social Science (ref)	1.000			1.000		
		Commerce	0.336	(0.176, 0.640)	0.001***	0.344	(0.144, 0.820)	0.016**
		Science	0.396	(0.244, 0.642)	0.000***	0.674	(0.417, 1.671)	0.246
**Socioeconomic status**								
	***Education of father***							
		Primary (≤ Class V) (ref)	1.000			1.000		
		Secondary (Class VI–Class X)	1.800	(0.919, 3.524)	0.087	2.504	(0.952, 6.589)	0.063
		Higher secondary (Class XI–Class XII)	0.666	(0.359, 1.235)	0.197	0.690	(0.254, 1.879)	0.468
		Tertiary (≥ Class XIII)	0.991	(0.571, 1.719)	0.973	2.663	(0.986, 7.192)	0.053
	***Occupation of father***							
		Unskilled/semi-skilled (ref)	1.000			1.000		
		Business	0.384	(0.214, 0.690)	0.001***	0.347	(0.138, 0.877)	0.025**
		Public/private service	0.403	(0.234, 0.694)	0.001***	0.371	(0.132, 1.042)	0.060
	***Income of father (in BDT)***							
		≤ 20,000 (ref)	1.000			1.000		
		20,001–40,000	0.705	(0.429, 1.159)	0.168	0.619	(0.277, 1.383)	0.242
		≥ 40,001	0.290	(0.162, 0.520)	0.000***	0.421	(0.176, 1.010)	0.053
	***Education of mother***							
		Primary (≤ Class V) (ref)	1.000			1.000		
		Secondary (Class VI–Class X)	1.000	(0.515, 1.941)	1.000	1.913	(0.895, 4.092)	0.094
		Higher secondary (Class XI–Class XII)	2.420	(1.295, 4.523)	0.006***	1.111	(0.495, 2.497)	0.799
		Tertiary (≥ Class XIII)	1.143	(0.587, 2.225)	0.694	1.788	(0.635, 5.029)	0.241
	***Occupation of mother***							
		Housewife (ref)	1.000			1.000		
		Working mother	0.336	(0.189, 0.596)	0.000***	0.605	(0.101, 3.618)	0.582
	***Income of mother (in BDT)***							
		No income (ref)	1.000			1.000		
		≤ 20,000	0.612	(0.306, 1.224)	0.165	0.979	(0.206, 4.645)	0.979
		≥ 20,001	0.165	(0.071, 0.380)	0.000***	0.632	(0.077, 5.190)	0.670
	***Type of family***							
		Extended (ref)	1.000			1.000		
		Nuclear	0.578	(0.354, 0.944)	0.028**	0.667	(0.333, 1.337)	0.254
	***Size of family (in Person)***							
		≤ 4 (ref)	1.000			1.000		
		≥ 5	1.581	(1.027, 2.433)	0.037**	1.469	(0.743, 2.903)	0.269
**Career choice index**								
		Job prospects	1.293	(1.215, 1.375)	0.000***	1.251	(1.161, 1.347)	0.000***
		Family preference	1.159	(1.068, 1.257)	0.000***	1.238	(1.099, 1.394)	0.000***
		Work diversity	0.947	(0.881, 1.017)	0.113	0.879	(0.795, 0.972)	0.012**

^***.^ Significant at 0.01%

^**.^ Significant at 0.05%

**COR** = Crude odds ratio; **AOR** = Adjusted odds ratio; **CI** = Confidence interval.

The adjusted logistic model indicates that sex, religion, educational track, occupation of the father as well as job prospects, family aspects, and work diversity, have played a decisive role in selecting relevant jobs by the university students. Findings reveal that male students were less likely to choose public/government jobs than their female counterparts (AOR: 0.281; 95% CI: 0.144 to 0.547). Muslim students, however, were 3.648 times more interested in pursuing a career in government organizations than students from *Sanatan* religion (AOR: 3.648; 95% CI: 1.765 to 7.542). Students with commerce or business studies background were more interested in the private sector for getting a job (AOR: 0.344; 95% CI: 0.144 to 0.820) than their compatriots with arts and social science as well as science backgrounds. The odds of selecting public jobs were 0.347 times (AOR: 0.347; 95% CI: 0.144 to 0.820) lower among students whose fathers were businessmen or entrepreneurs. Students, who prioritized job facilities as well as their families, were 1.251 times (AOR: 1.251; 95% CI: 1.161 to 1.347) and 1.238 times (AOR: 1.238; 95% CI: 1.099 to 1.394) more likely to select public jobs. On the contrary, those who emphasized on more dynamic and challenging work were 0.879 times (AOR: 0.879; 95% CI: 0.795 to 0.972) less likely to choose government jobs. It is, therefore, apparent that students who wanted a stable job that allows the individual to spend sufficient time with respective families were more inclined to get government jobs.

## Discussion

This study aimed at identifying the career choices of university students, and the factors influencing their preferences. Considering Homans’ [25] view of ‘fair exchange’ together with Coleman’s [26] interpretation of ‘social capital’, several social, economic, and other relevant factors have been used to explain its association with career choices. Findings suggest that social capital–personal characteristics and socioeconomic background–as well as the expected ‘benefits’ are the key determinants to understand the career choices–public versus private–of public university students in Bangladesh.

Findings of this research indicate that male students preferred working in private sectors, while their female counterparts were more interested in public jobs. It has widely been recognized that men and women have different preferences relating to work and career, as the former placed a higher value on working outside in a more challenging environment while the latter expected to carry out domestic duties within household, whether in a conservative [19] or a liberal society [39]. In patriarchal societies, women generally select socially accepted feminine occupations, such as teaching or other desk jobs, which ensure secured career, stable salary as well as negotiable workload to spend time with family [12, 48]. Men, on the other hand, traditionally carry family liabilities, such as to provide financial support to parents, and sometimes, siblings, hence, they are often compelled to seek challenging or complicated yet high paid tasks [39] in private sectors in Bangladesh due to better salary and professional development [24]. It is also important to note that the public jobs in Bangladesh generally require a longer time to get appointed, which may have motivated young males to select private employment over the public ones. The issue, however, demands an in-depth inquiry in detail, which this study could not address.

Religious beliefs have significant relation with career choices as orthodox religious spirituality, especially among Abrahamic religions, often inspire people to get interested in non-profit and faith-based humanitarian agencies to achieve spiritual rewards [21, 41]. Like other Abrahamic religious sects, Muslims often choose their occupations to help and support human beings as well as to promote good things in life [21]. This study found that Muslim students were more likely to choose public jobs rather than private ones. This tendency could be explained by Flanigan’s [21] observation that Muslims, following the principles of Islam, often took the opportunities to serve people in public jobs compared to other religious sects. It is, however, undeniable that the role of ethical obligations in selecting career paths has remained unexplained in this study; thus, the authors recommend an inductive approach to unearth the underlying spiritual issues in making career decisions.

The educational track also influences students’ career choices. This study suggests that the students with commerce background preferred private jobs over the public ones. This finding is coherent with previous studies that students with business studies experience are more aware of career goals, and they have a competitive advantage in management skills over students of other disciplines, such as arts and science [49]. Furthermore, from an ideological legacy, business and management studies promote management skills and knowledge among its graduates and suggest the students to consider business and organizational management as professional careers, a faster route to managerial jobs, with extensive knowledge and information regarding possible barriers and impediments [50].

Apart from personal attributes, family background plays a crucial role in determining a career for young people. Fouad, Cotter [33] and Kaneez and Medha [37], for example, observed that parental education, as well as their expectations, extensively influence students’ views on whether to continue education or pursue desired professions. Likewise, Moakler Jr. and Kim [38] claimed that students often follow the ‘footprints’ of their parents to pursue similar careers. The findings of this study suggest that paternal occupation, particularly the entrepreneurship, was significantly associated with the preference of private jobs, thereby complemented previous studies. In Bangladesh, parents, in general, motivate their children to get a job that pays a ‘sizable’ amount [24]. Moreover, they often encourage children to ‘inherent’ [51] the occupations of their parents or an ‘unrealized’ career that parents often ‘fantasized’ when they were young [48].

The significance of personal and socioeconomic background in selecting career paths is, indeed, undeniable. Some other unexplored factors have also been investigated to understand the dilemma between public and private jobs. Findings suggest that ‘job prospects’ and ‘family preferences’ were positively associated with public services, and the ‘work diversity’, on the other hand, was significantly associated with private jobs. A recent study in Bangladesh suggests that young men and women have placed great importance on extrinsic aspects when selecting careers, such as asset accumulation, social and economic security [12]. Similarly, this study shows that public services were positively related to job prospects as well as its intrinsic facilities, such as limited working hours to meet family responsibility. Previous studies show that individuals’ career decisions have largely been influenced by job prospects and opportunities, including monetary incentives, promotion, and professional development [17–19] followed by a balance between work and family [36]. The latter, however, has primarily been prioritized by female students [35, 36, 39], and such would allow the intellectual satisfaction through work in the limited hour [19, 36]. On the contrary, private jobs had a positive relation with ‘job diversity’ that would allow an individual to get involved in a highly demanding job, characterized by ‘hovering’ around the country and sometimes the world, to explore new challenges and opportunities. Earlier studies also indicate that some people are interested in jobs that offer challenges and other specific job characteristics compelling individuals to work under pressure [19, 29, 30].

Despite some exciting findings, the authors suggest readers’ discretion to interpret the findings of this study. Generalization of the results is limited to the students at a public university only. The socioeconomic background was also not controlled. Moreover, the non-response from selected students may indulge a sample biasness; therefore, it may not be entirely possible to understand whether the career choices made by the participants are exclusively determined by sex, religion, educational track, or other socioeconomic aspects. Regardless of these limitations, a decent estimation through rigorous statistical analysis to explain the reasons behind career choices provides a ground to understand the career dynamics of university students in Bangladesh.

## Conclusion

This study focused on determining the career choices made by university students in Bangladesh and identifying the factors influencing their career choices, incorporating Homans’ and Coleman’s theoretical perspectives. The findings indicate public/government jobs were highly prioritized by young Bangladeshi people, however, the sex and religious identity followed by educational track and paternal occupation were the most influential factors in setting a career goal. As already described, students preferring job prospects and family responsibility selected public services; the work diversity, on the other hand, was associated with private jobs. Nevertheless, this study provided invaluable information regarding the career dilemma among university students and unearthed the determinants of career choice decisions. Not only these findings are a significant contribution to the existing literature on career choices of university students in the context of Bangladesh, but also this information, without any doubt, is essential for policymakers to (re)-think and (re)-consider the explored factors when offering jobs, public/government, or private/non-government. Nonetheless, researchers also anticipate that it is vital for higher educational institutions as well as the government to analyze the supply-demand chain and to re-design academic curriculum to keep things aligned for future job specifications.
